# Prevalence of Central Sensitization in Postural Tachycardia Syndrome

**DOI:** 10.1001/jamanetworkopen.2025.53694

**Published:** 2026-01-13

**Authors:** Gabrielle T. Mathew, Peter Novak

**Affiliations:** 1 Department of Neurology, Mass General Brigham, Boston, Massachusetts; 2 Harvard Medical School, Boston, Massachusetts

## Abstract

**Importance:**

A previous study showed a high prevalence of central sensitization syndrome (CSS) in patients with autonomic symptoms. The prevalence of CSS in postural tachycardia syndrome (POTS), a form of dysautonomia, is unknown.

**Objectives:**

To analyze the prevalence of CSS in POTS.

**Design, Setting, and Participants:**

This case-control study included patients with a POTS diagnosis confirmed by autonomic testing at Brigham and Women’s Faulkner Hospital between 2022 and 2025. Data were analyzed from April to August 2025.

**Exposure:**

POTS with and without CSS.

**Main Outcomes and Measures:**

Central Sensitization Inventory (to assess central sensitization syndrome [CSS]), COMPASS-31 (autonomic symptoms), Neuropathy Total Symptom Score-6 (NTSS-6, sensory symptoms), PROMIS (global health), and autonomic testing (Valsalva maneuver, deep breathing, sudomotor function, and head-up tilt) with skin biopsies. Primary outcome was the central sensitization inventory score with secondary outcomes individual test performances.

**Results:**

This study included 305 patients with POTS, of whom 264 (86.6%) met criteria for CSS (mean [SD] age, 33.21 [10.75] years; 30 males [11.4%]; 234 females [88.6%]). Patients with CSS compared with those without CSS had longer duration of symptoms, were more frequently female, exhibited higher rates of anxiety (195 [73.9%] vs 20 [48.8%]; *P* = .002), depression (168 [63.6% vs 14 [34.1%]; *P* = .001), fibromyalgia (46 [17.4%] vs 0 [0%]; *P* = .008), irritable bowel syndrome (IBS, 90 [34.1%] vs 7 [17.1%]; *P* = .046), headaches (176 [66.7%] vs 12 [29.3 %]; *P* < .001), treatment with antihistamine medication (136 [51.5%] vs 13 [31.7%]; *P* = .03), psychiatric medication (163 [61.7%] vs 17 [41.5 %]; *P* = .02), pain medication (127 [48.1%] vs 8 [19.5%]; *P* = .001), and gastrointestinal medication (82 [31.1%] vs 5 [12.2 %]; *P* = .02), and had higher COMPASS-31 scores (51.93 [13.23] vs 31.18 [10.49]; *P* < .001), NTSS-6 scores (11.32 [4.86] vs 4.44 [3.32]; *P* < .001), NRS scores (3.26 [2.73] vs 0.54 [1.21]; *P* < .001), and worse PROMIS scores (20.36 [5.45] vs 27.96 [4.73]; *P* < .001). Autonomic tests showed lower orthostatic end-tidal carbon dioxide (27.59 [6.39] mm HG vs 29.46 [4.68] mm HG; *P* = .002) and a greater orthostatic decline in cerebral blood flow velocity (17.08 [8.72] cm/sec vs 13.68 [5.04] cm/sec; *P* < .001) in the CSS group. Both groups had similar prevalence of autonomic failure (223 [84.5%] vs. 33 [80.5%]; *P* = .67, mostly mild intensity), and abnormal skin biopsy (43% in both groups).

**Conclusions and Relevance:**

These findings suggest that CSS was common in patients with POTS and may represent a higher-order sequela of cerebrovascular, respiratory, and autonomic dysregulation. This heightened central processing may amplify symptom perception through altered interoceptive signaling. Central sensitization and autonomic impairment may coexist, and management should focus on both conditions.

## Key Points

**Question:**

What is the prevalence of central sensitization syndrome in postural tachycardia syndrome (POTS)?

**Findings:**

This case-control study of 305 patients found a high prevalence (86.5%) of central sensitization syndrome in individuals with POTS.

**Meaning:**

These findings suggest that co-occurring central sensitization syndrome may exacerbate the disease burden in POTS; enhancing knowledge of this comorbidity could lead to more precise and comprehensive diagnostics and treatment strategies.

## Teaser

This case-control study analyzes the prevalence of central sensitization syndrome in patients with postural tachycardia syndrome among those presenting at a single academic medical center.

## Introduction

Patients with postural tachycardia syndrome (POTS) often present with a broad spectrum of chronic symptoms that suggest autonomic dysfunction, including orthostatic intolerance, fatigue, cognitive disturbances, gastrointestinal complaints, and diffuse pain.^1^ Despite significant clinical impairment, standard autonomic testing often reveals only mild or limited abnormalities.^2^ This disconnect between symptom severity and objective physiological findings presents a persistent challenge in both diagnosis and treatment.^3,4^

One proposed explanation for this discrepancy is central sensitization, a condition involving heightened central nervous system responsiveness.^3^ Many patients with chronic autonomic complaints exhibit features of central sensitization, which are presumably associated with altered interoceptive processing, which may amplify symptom perception.

In this study, we investigated the central sensitization as a potential contributor to symptom burden in patients with POTS. We aimed to assess the prevalence of central sensitization and its association with symptom severity and objective autonomic findings.

## Methods

This retrospective case-control single-center study included patients who underwent autonomic testing at the Brigham and Women’s Faulkner Hospital Autonomic Laboratory, Boston, between 2022 and 2025. The medical information was obtained from the patient’s electronic records. This case-control study followed the Strengthening the Reporting of Observational Studies in Epidemiology (STROBE) reporting guideline.

The institutional review board of Brigham and Women’s Hospital approved the study as a minimal-risk study, and the consent form signature was waived. The study includes patients referred for autonomic testing to evaluate chronic (eg, 6 months or more) orthostatic intolerance (OI), in which the testing was consistent with POTS. OI was defined as the presence of symptoms of cerebral hypoperfusion with standing and relief of symptoms by recumbency.^1^ The most common orthostatic symptom is lightheadedness or dizziness accompanied by dyspnea. POTS was defined as a combination of OI and orthostatic tachycardia (30 or more beats per minute in those older than 19 years or 40 or more beats per minute for adolescents without orthostatic hypotension).^5^ Exclusion criteria were history of pulmonary, cardiac disorder, and metabolic derangement, and other mimickers of POTS,^1^ inability to complete surveys, inability to hold medication that interferes with autonomic function and inability to complete autonomic testing.

### Patient-Reported Surveys

Patients fill out validated surveys (eMethods in Supplement 1), including Central Sensitization Inventory (CSI)^6^ (to assess central sensitization syndrome [CSS] defined as CSI, part A score 40 or more), COMPASS-31^7^ (to assess autonomic symptoms), Neuropathy Total Symptom Score-6^8^ (NTSS-6, to assess sensory symptoms) using the 11-point Numerical Rating Scale (NRS to assess pain intensity^9^ and PROMIS^10^ (to assess global health).

### Autonomic Tests

The Brigham protocol (eMethods in Supplement 1) was used to assess autonomic functions and small fiber neuropathy.^11^ The tests included deep breathing (a marker of parasympathetic cardiovagal functions), the Valsalva maneuver and the head-up tilt test (both markers of parasympathetic and adrenergic sympathetic functions), and sudomotor evaluation (a marker of postganglionic sudomotor functions). Epidermal nerve fiber density (ENFD) and sweat gland nerve fiber density (SGNFD) were used to assess the presence of neurodegeneration of small fibers using established standards.^12^ Test results were graded using the Quantitative Scale for Grading of Cardiovascular Autonomic Reflex Tests and Small Fibers from Skin Biopsies (QASAT).^11^

### Statistical Analysis

CSS (ie, CSI of 40 or more) and non–CSS (CSI of less than 40) groups were compared using the 2-sample *t* test for continuous variables and the χ^2^ test for categorical variables. Missing data were ignored. Repeated-measures design with a linear mixed-effects model has been used to evaluate the effect of diagnosis on orthostatic hemodynamic variables adjusted for baseline. We used the formula:

fit<-lmer(hemvar ~ orthostasis + diagnosis + (1|patient); where hemvar is the hemodynamic variable (heart rate, blood pressure, cerebral blood flow velocity [CBFv], end-tidal carbon dioxide [CO_2_]), orthostasis equals the head-up tilt duration, diagnosis equals CSS or non–CSS and the patient was a random effect. CBFv using the percentage difference from supine baseline, while absolute values were used for the remaining hemodynamic variables in the linear model formula.

Statistical analyses were done using R version 4.1 (R Project for Statistical Computing), using the lmerTest^13^ and ggstatsplot^14^ packages. Data were analyzed from April to August 2025. Statistical significance was set as *P* < .05, and all tests were 2-sided.

## Results

From a total of 1195 successive patients who underwent autonomic testing, 305 patients satisfied the POTS criteria and were analyzed (Table 1). The mean (SD) CSI score was 57.6 (15.4), and 264 patients (86.6%) met the criteria for CSS (mean [SD] age, 33.21 [10.75] years; 30 males [11.4%]; 234 females [88.6%]). Patients with CSS were of similar age, had longer symptom duration (mean [SD], 7.79 [6.88] years vs 5.48 [4.93] years; *P* = .047) and were more frequently females as compared with those without CSS. The CSS group had more frequent anxiety (195 [73.9%] vs 20 [48.8%]; *P* = .002), depression (168 [63.6% vs 14 [34.1%]; *P* = .001), fibromyalgia (46 [17.4%] vs 0;* P *= .008), irritable bowel syndrome (IBS, 90 [34.1%] vs 7 [17.1%]; *P* = .046]), and headaches (176 [66.7%] vs 12 [29.3 %]; *P* < .001) and were treated more often with anti-histamine (136 [51.5%] vs 13 [31.7%]; *P* = .03), psychiatric (163 [61.7%] vs 17 [41.5 %]), pain (127 [48.1%] vs 8 [19.5%]; *P* = .001), and gastrointestinal (82 [31.1%] vs 5 [12.2 %]; *P *= .02) medication.

**Table 1.** Demographic and Baseline Characteristics

Variable	Patients, No (%)		*P* value^a^
	CSS (n=264)	Non–CSS (n=41)	
Age, years	33.21 (10.75)	32.78 (10.39)	.81
Gender			
Male	30 (11.4)	11 (26.8)	.02
Female	234 (88.6)	30 (73.17)	
BMI	25.34 (6.10)	24.75 (4.74)	.55
Symptoms duration, mean (SD), years	7.79 (6.88)	5.48 (4.93)	.047
Comorbidities			
Long COVID	34 (12.9)	6 (14.6)	.95
Myalgic encephalomyelitis/ chronic fatigue syndrome	20 (7.6)	4 (9.8)	.86
Depression	168 (63.6)	14 (34.1)	.001
Fibromyalgia	46 (17.4)	0	.008
Irritable bowel syndrome	90 (34.1)	7 (17.1)	.046
Anxiety	195 (73.9)	20 (48.8)	.002
Headaches	176 (66.7)	12 (29.3)	<.001
Medical therapy			
Antihistamine	136 (51.5)	13 (31.7)	.03
Pain	127 (48.1)	8 (19.5)	.001
Pressor^b^	81 (30.7)	12 (29.3)	.99
Psychiatric	163 (61.7)	17 (41.5)	.02
Hypertension	26 (9.8)	1 (2.4)	.21
Antitachycardic^c^	67 (25.4)	7 (17.1)	.34
Gastrointestinal	82 (31.1)	5 (12.2)	.02
Immunomodulators	6 (2.3)	0	.71

Abbreviations: BMI, body mass index (calculated as weight in kilograms divided by height in meters squared); CSS, central sensitization syndrome.

^a^ Calculated using *t* test or χ^2^ test as appropriate.

^b^ Includes fludrocortisone, proamatine, pyridostigmine, and droxydopa.

^c^ Includes adrenergic β-blockers, calcium channel blockers, and ivabradine.

### Surveys

All patient-reported surveys were consistent with a higher symptom burden in CSS. Patients with CSS had higher mean (SD) scores on Compass-31 (51.93 [13.23] vs 31.18 [10.49]), NTSS-6 (11.32 [4.86] vs 4.44 [3.32]), NRS (3.26 [2.73] vs 0.54 [1.21]), and PROMIS global health (20.36 [5.45] vs 27.96 [4.73]) (*P* < .001) compared with patients without CSS (Table 2).

**Table 2.** Patients Reported Outcome Measures

Domain	Patients, mean (SD)		*P* value^a^
	CSS (n = 264)	Non–CSS (n = 41)	
Compass-31 scores			
Total	51.93 (13.23)	31.18 (10.49)	<.001
Orthostatic	26.64 (7.71)	19.22 (8.31)	<.001
Vasomotor	2.52 (1.58)	1.46 (1.58)	<.001
Secretomotor	5.92 (3.85)	2.04 (3.07)	<.001
Gastrointestinal	11.82 (4.25)	6.58 (3.64)	<.001
Urinary	2.24 (2.19)	0.54 (1.17)	<.001
Pupillomotor	51.93 (13.23)	31.18 (10.49)	<.001
NTSS-6 scores			
Total score	11.32 (4.86)	4.44 (3.32)	<.001
Aching frequency	2.63 (0.66)	1.59 (1.09)	<.001
Aching intensity	1.97 (0.76)	1.00 (0.77)	<.001
Allodynia frequency	1.16 (1.24)	0.29 (0.72)	<.001
Allodynia intensity	0.85 (1.00)	0.22 (0.57)	<.001
Burning frequency	1.66 (1.16)	0.54 (0.98)	<.001
Burning intensity	1.33 (1.07)	0.37 (0.73)	<.001
Lancinating frequency	1.91 (1.03)	0.68 (0.96)	<.001
Lancinating intensity	1.74 (1.06)	0.61 (0.97)	<.001
Prickling frequency	2.24 (0.89)	1.22 (1.13)	<.001
Prickling intensity	1.58 (0.83)	0.68 (0.69)	<.001
Numbness frequency	2.15 (1.02)	1.05 (1.12)	<.001
Numbness intensity	1.52 (0.90)	0.61 (0.70)	<.001
Pain, numerical rating scale	3.26 (2.73)	0.54 (1.21)	<.001
PROMIS Scale v1.2 scores			
Physical health	9.87 (2.76)	13.54 (3.13)	<.001
Mental health	10.49 (3.25)	14.43 (2.36)	<.001
Global health	20.36 (5.45)	27.96 (4.73)	<.001

Abbreviations: CSS, central sensitization syndrome; NTSS-6, Neuropathy Total Symptom Score-6.

^a^ Calculated using the *t* test.

### Autonomic Testing

There was no difference in deep breathing and Valsalva maneuver comparing the CSS group and non–CSS group (eTable 1 and eFigure 1 in Supplement 1). In the supine position, the CSS group compared with the non–CSS group had a higher heart rate (76.64 [12.98] beats per minute vs 70.58 [14.48] beats per minute; *P* = .007), diastolic CBFv (45.47 [10.25] cm/sec vs 42.43 [7.02] cm/sec; *P* = .02), and lower end-tidal CO_2_ (34.26 [3.79] mm HG vs [35.73 [3.91] mm HG; *P* = .02). During the head-up tilt (Figure and eFigure 1 in Supplement 1), patients with CSS vs those without CSS had a higher heart rate (110.27 [18.14] beats per minute vs 99.59 [15.96] beats per minute; *P* < .001), systolic blood pressure (116.16 [14.75] mm HG vs 109.56 [12.62] mm HG; *P* = .007), mean blood pressure (91.54 [11.76] mm HG vs 86.51 [10.42] mm HG; *P* = .01), diastolic blood pressure (79.22 [11.29] mm HG vs 74.98 [9.7] mm HG; *P* = .02), respiratory frequency (16.04 [6.71] breaths per minute vs 13.29 [4.94] breaths per minute; *P* = .003), lower end-tidal CO_2_ (27.59 [6.39] mm HG vs 29.46 [4.68] mm HG; *P* = .03) and greater decline in mean CBFv (17.08 [8.72] cm/sec vs 13.68 [5.04] cm/sec; *P* < .001).

**Figure.**
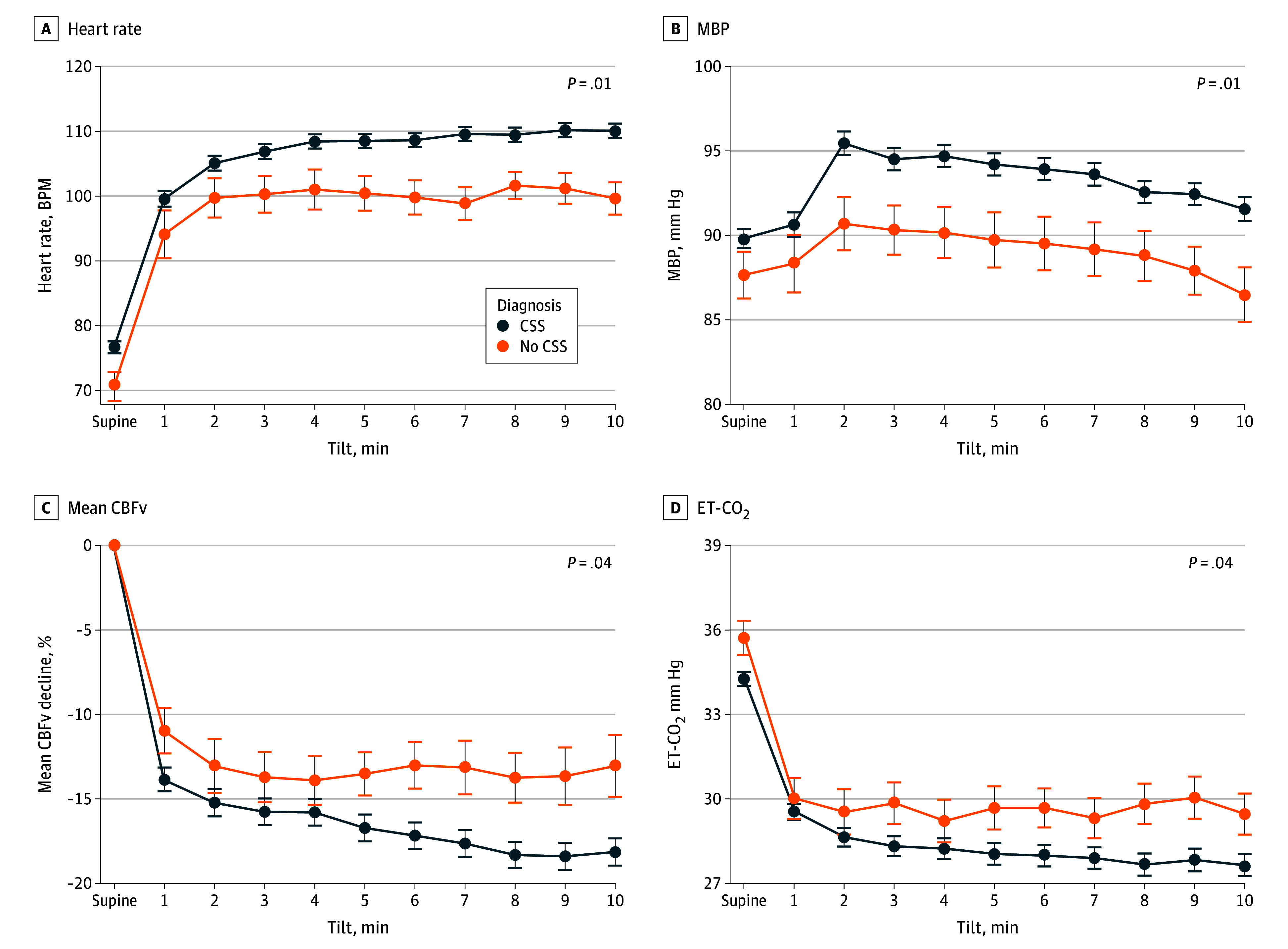
The Head-Up Tilt Test Profile Data are hemodynamic variables at supine baseline and at every minute of the tilt, with points indicating the mean and error bars indicating 95% CIs. A significant association of diagnosis (CSS vs no CSS) with the orthostatic responses using a linear mixed-effects model was noted for all hemodynamic variables; *P* value indicates the model effect. CBFv, cerebral blood flow velocity; CSS, central sensitization syndrome; ET-CO_2_, end-tidal CO_2_; MBP, mean blood pressure.

The CSS group compared with the non–CSS group had higher (ie, more abnormal) QASAT-CBFv scores grading orthostatic decline of CBFv (6.34 [3.29] vs 4.71 [3.0]; *P* = .003) (eTable 1 in Supplement 1). Autonomic failure, most commonly of mild grade, was detected in both groups at a similar frequency. Abnormal findings on skin biopsies had similar prevalence in both groups (eTable 1 in Supplement 1).

Linear models revealed a significant effect of diagnosis (CSS vs non–CSS) on orthostatic responses (Figure 1). Specifically, individuals with CSS exhibited a greater decline in mean orthostatic CBFv (β = 3.21; 95% CI, 0.19 to 6.24; *P* = .04), lower orthostatic end-tidal CO_2_ (β = 1.66; 95% CI, 0.04 to 3.27; *P* = .04), higher orthostatic heart rate (β = −7.77; 95% CI, −13.03 to −2.50; *P* = .01), and elevated orthostatic mean blood pressure (β = −4.05; 95% CI, −7.21 to −0.88; *P* = .01), compared with those in the non–CSS group.

## Discussion

This study suggests that central sensitization may be common among patients with POTS, as 86.6% of our cohort met the criteria for CSS. These patients had a higher burden of global health, autonomic and sensory symptoms, more pain, and they exhibited objective physiological differences, namely greater orthostatic decline in orthostatic CBFv, lower orthostatic end-tidal CO_2_, and exaggerated orthostatic tachycardic and pressor responses.

These findings highlight that CSS is not merely a comorbid condition but potentially a downstream consequence of the complex cerebrovascular, respiratory, and autonomic perturbations intrinsic to POTS. Specifically, these results show strong links between CSS and symptom severity. The increased symptom burden seen in patients with POTS and CSS can be attributed to heightened central nervous system sensitivity to autonomic and sensory stimuli due to disrupted interoceptive processing pathways.^3,15,16^ Enhanced interoception increases awareness of internal body sensation, such as heartbeat, vasomotor, and other autonomic activities. CSS has been linked to neuroinflammation in the brain and spinal cord^17^ characterized by microglial activation and the release of proinflammatory mediators.^18^ Imaging studies showed functional alterations in neural circuits that also regulate autonomic function, such as the anterior cingulate cortex, insula, and brainstem.^19^ As key regions in interoceptive processing, the anterior cingulate and insula,^19,20^ may cause faulty processing of autonomic signals with a net effect of amplifying the symptoms burden. According to this concept neuroinflammation associated with central sensitization and altered proprioception may help explain the disproportionate symptom burden observed in the CSS group despite relatively mild abnormalities on standard autonomic testing. The validity of this concept can be tested by targeting neuroinflammation and/or interoception.^21^

Cerebrovascular and respiratory dysregulation, which were disproportionately higher in the CSS group, further supported the presence of central nervous system dysfunction. Previous studies have shown that patients with POTS exhibit reduced CBFv, likely associated with cerebral arteriolar vasoconstriction induced by hypocapnic hyperventilation.^5,22^ Reduced orthostatic CBFv in POTS has been associated with symptoms of cerebral hypoperfusion, such as orthostatic lightheadedness.^22,23,24^ In our study, the more symptomatic CSS group had lower orthostatic CBFv and end-tidal CO_2_ levels, consistent with a greater degree of cerebral hypoperfusion. Cerebral hypoperfusion may represent an additional factor contributing to the development of central sensitization,^25^ as it can lead to cerebral hypoxia^26^ which sensitizes central nervous system neurons.^27^

Demographic characteristics of our CSS group are also consistent with features commonly associated with central sensitization, such as a longer symptom duration^28^ and predominance of female patients.^1,6^ Furthermore, the CSS group exhibited a higher prevalence of comorbid conditions linked to central sensitization, such as depression,^29^ anxiety,^29^ fibromyalgia,^30^ IBS,^31^ and headaches.^28^ This group also showed more frequent use of chronic pain medications, further supporting the association with central sensitization.^28^

The coexistence of autonomic impairment and CSS highlights the limitations of treatment strategies that focus only on hemodynamic stabilization. While volume expansion, compression garments, or pharmacologic modulation of heart rate and blood pressure remain essential, our data suggested that symptom relief may not be complete unless central mechanisms were also addressed.

### Limitations

This study has limitations. Its retrospective design limits the ability to draw causal inferences. Additionally, the single-center nature of the study and the potential for referral bias may have influenced the characteristics of the cohort, as patients at this center may not fully represent the broader population. Together, these limitations warrant cautious interpretation of the results, particularly regarding the generalizability of the findings.

## Conclusions

These findings suggest that central sensitization may be a common and clinically important component of POTS, closely linked to symptom severity and measurable cerebrovascular-respiratory instability. Recognizing this interplay could help establish more effective and comprehensive management strategies, with the overarching goal of improving the quality of life for individuals affected by POTS. Nevertheless, further studies are needed to confirm our findings.

## Notes

Supplement 1.
eMethods.
**eFigure. **QASAT **eTable 1. **Results of Autonomic Testing **eTable 2. **Frequency of Abnormal Tests

Supplement 2.
Data Sharing Statement
