# Supplementary material for: Prevalence of Central Sensitization in Postural Tachycardia Syndrome
Source: JAMA Netw Open. 2026 Jan 13;9(1):e2553694. doi: 10.1001/jamanetworkopen.2025.53694 (PMC12801080; doi:10.1001/jamanetworkopen.2025.53694)
Supplement: Supplement 1. — eMethods. eFigure. QASAT eTable 1. Results of Autonomic Testing eTable 2. Frequency of Abnormal Tests [file jamanetwopen-e2553694-s001.pdf]

Supplemental Online Content

Matthew GT and Novak P. Prevalence of central sensitization in postural tachycardia syndrome (POTS). *JAMA Netw Open*. 2025;9(1):e2553694. doi:10.1001/jamanetworkopen.2025.53694

eMethods.

eFigure. QASAT

eTable 1. Results of Autonomic Testing

eTable 2. Frequency of Abnormal Tests

This supplemental material has been provided by the authors to give readers additional information about their work.

© 2025 Matthew GT et al. *JAMA Network Open*.

eMethods

Patient-reported surveys

Patients filled out validated surveys, including the central sensitization inventory[1] (CSI), Compass-31[2], Neuropathy Total Symptom Score-6[3] (NTSS) and the NIH Toolbox pain-intensity[4].

CSI is a validated instrument for assessment of the central sensitization. CSI part A, scores on a 5-point Likert scale (0=never, 4=always), has a range of 0 - 100. A cut-off score of 40 is used for the differentiation of central sensitization syndrome (CSS).

Compass-31 is a validated general instrument for the quantification of autonomic symptoms. Compass-31 assesses autonomic symptom severity in six domains (orthostatic, vasomotor, secretomotor, gastrointestinal, bladder, and pupillomotor) over the last year. Scores range from 0 to 100, with higher scores representing more severe symptoms.

Sensory complaints and pain over the preceding 24 hours were assessed using the validated Neuropathy Total Symptom Score-6 (NTSS). NTSS quantifies sensory symptoms into six types: numbness and/or insensitivity, prickling and/or tingling, burning sensation, aching pain and/or tightness, sharp, shooting, lancinating pain; and allodynia and/or hyperalgesia. The NTSS range is 0-21.96, and the severity and frequency of sensory symptoms is proportional to the NTSS score.

The pain intensity for the previous seven days was assessed using the 11-point Numerical Rating Scale (NRS).

Global physical and mental health was assessed by a PROMIS Scale 1.2, a validated instrument measuring health status.

Autonomic tests

The Brigham protocol was used to objectively assess autonomic functions and small fiber neuropathy (SFN). Patients were instructed to abstain from alcohol, smoking and caffeine for at least 24 hours, and from food for at least two hours prior to testing. Medications affecting the

© 2025 Matthew GT et al. *JAMA Network Open*.

autonomic function were discontinued for five half-lives or longer before the testing. We have previously described the protocol in detail.[5,6] Briefly, cardiovascular functional autonomic testing included deep breathing (a marker of parasympathetic cardiovagal functions), the Valsalva maneuver and the head-up tilt test (both markers of parasympathetic and adrenergic sympathetic functions), and sudomotor evaluation (a marker of postganglionic sudomotor functions). Patients were rested for 10 minutes in the supine position and then were tilted upright at 70 degrees for 10 minutes. Continuously recorded signals were electrocardiogram, blood pressure, respiratory movement, end-tidal CO_2, respiratory rate, and CBFv in the middle cerebral artery using Transcranial Doppler using MultiDop T (Multigon, New York). Blood pressure was obtained intermittently using an automated oscillometric blood pressure monitor Welch Allyn CVSM 6400 Monitor (Skaneateles Falls, NY) from the arm and continuously using the photoplethysmographic signal volume-clamped by the servo control (NIBP Nano, ADInstruments, Sydney, Australia) in the finger. An electrochemical skin conductance (ESC)[7] was used to evaluate the sudomotor functions using the Sudoscan device (Impeto Medical, Paris, France).

Skin biopsies

Epidermal nerve fiber density (ENFD) and sweat gland nerve fiber density (SGNFD) were used to assess the presence of neurodegeneration of small fibers.[8] Using established standards, the skin samples were taken from the proximal thigh and calf using a circular 3-mm punch tool. The samples were immunostained with a pan-neuronal marker PGP 9.5. The processing was done at Therapath (New York, NY). Normative data are age-and gender dependent and were published previously.[9]

Grading of Small Fiber Neuropathy, Dysautonomia, and Cerebral Blood Flow

Test results were graded using the Quantitative Scale for Grading of Cardiovascular Autonomic Reflex Tests and Small Fibers from Skin Biopsies (QASAT).[5] QASAT is an objective instrument for grading the severity of dysautonomia, small fiber neuropathy, and cerebral blood flow

© 2025 Matthew GT et al. *JAMA Network Open*.

abnormalities. Each domain is scored, where a score equal to 0 is normal, and above 0 is abnormal with the severity proportional to the number. In this study, we used the following QASAT scores:

QASAT-Autonomic failure =QASAT-Cardiovagal + QASAT-Adrenergic + QASAT-Sudomotor. QASAT-Autonomic failure score has a range of 0-22, and moderate failure (score >3) is clinically significant. QASAT-Cardiovagal score (range 0-3) was obtained from deep breathing test, QASAT-Adrenergic score was obtained from Valsalva maneuver and the head-up tilt test as follows:

QASAT-Adrenergic (range 0-13) = QASAT-Valsalva maneuver (range 0-3) + QASAT-Orthostatic hypotension (range 0-10). QASAT-Sudomotor was obtained from the ESC (range 0-6). QASAT-ENFD (range 0-8) and QASAT-SGNFD (range 0-8) were obtained from skin biopsies. QASAT scores were calculated using the qpack package.[10]

Supplements reference

1. Neblett R, Hartzell MM, Mayer TG, Cohen H, Gatchel RJ. Establishing Clinically Relevant Severity Levels for the Central Sensitization Inventory. *Pain Pract Off J World Inst Pain*. 2017;17(2):166-175. doi:10.1111/papr.12440

2. Sletten DM, Suarez GA, Low PA, Mandrekar J, Singer W. COMPASS 31: A Refined and Abbreviated Composite Autonomic Symptom Score. *Mayo Clin Proc*. 2012;87(12):1196-1201. doi:10.1016/j.mayocp.2012.10.013

3. Bastyr EJ, Price KL, Bril V, MBBQ Study Group. Development and validity testing of the neuropathy total symptom score-6: questionnaire for the study of sensory symptoms of diabetic peripheral neuropathy. *Clin Ther*. 2005;27(8):1278-1294. doi:10.1016/j.clinthera.2005.08.002

4. Cook KF, Dunn W, Griffith JW, et al. Pain assessment using the NIH Toolbox. *Neurology*. 2013;80(11 Suppl 3):S49-S53. doi:10.1212/WNL.0b013e3182872e80

5. Novak, Peter. *Autonomic Testing*. Oxford University Press; 2019.

6. Novak P. Quantitative autonomic testing. *J Vis Exp JoVE*. 2011;(53). doi:10.3791/2502

7. Novak P. Electrochemical skin conductance: a systematic review. *Clin Auton Res Off J Clin Auton Res Soc*. 2019;29(1):17-29. doi:10.1007/s10286-017-0467-x

© 2025 Matthew GT et al. *JAMA Network Open*.

8. Devigili G, Tugnoli V, Penza P, et al. The diagnostic criteria for small fibre neuropathy: from symptoms to neuropathology. *Brain J Neurol*. 2008;131(Pt 7):1912-1925. doi:10.1093/brain/awn093

9. Novak P, Mukerji SS, Alabsi HS, et al. Multisystem Involvement in Post-acute Sequelae of COVID-19 (PASC). *Ann Neurol*. Published online December 24, 2021. doi:10.1002/ana.26286

10. Novak P. Qpack-a Python package for QASAT-quantitative scale for grading cerebral blood flow, autonomic testing, and skin biopsies. *Neurol Sci Off J Ital Neurol Soc Ital Soc Clin Neurophysiol*. 2022;43(8):4821-4828. doi:10.1007/s10072-022-06007-w

© 2025 Matthew GT et al. *JAMA Network Open*.

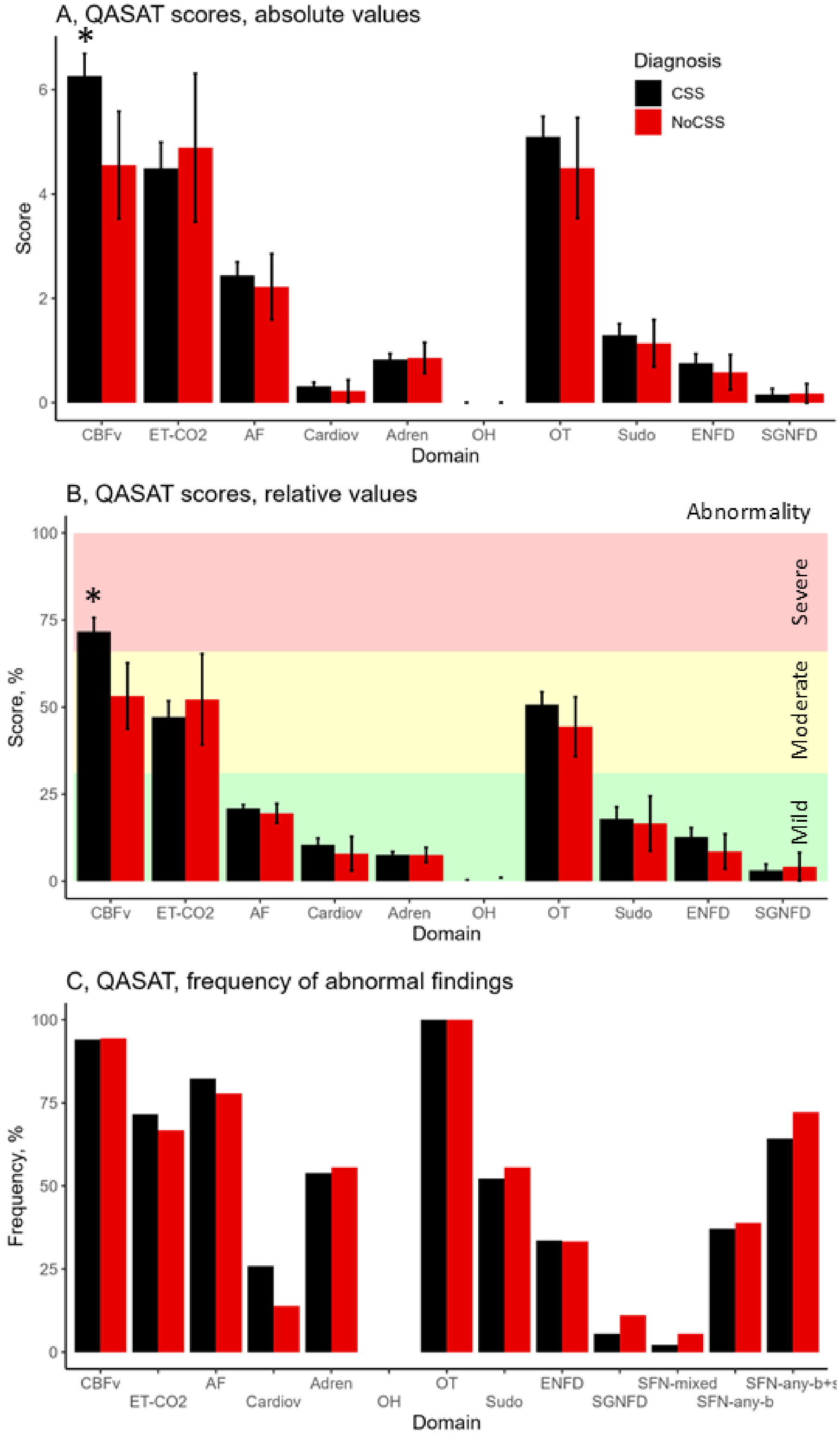

eFigure. QASAT Scores

A: Absolute scores, mean±conf.int, B: Relative scores in percent, mean±conf.int. Since the range of absolute QASAT scores differs for different domains, panel B shows scores adjusted for the 0-100% range, where 0=normal, 100% is a maximal abnormality, C: Percentage of patients in which the QASAT score was abnormal (>0). AF=autonomic failure, Cardiov=cardiovagal, Adren=adrenergic, OH=neurogenic orthostatic hypotension, OT=orthostatic tachycardia, Sudo=sudomotor, ENFD=epidermal nerve fiber density, SGNFD=sweat gland nerve fiber density.

© 2025 Matthew GT et al. *JAMA Network Open*.

eTable 1. Results of Autonomic Testing			
Variable	CSS (n=264)	Non - CSS (n=41)	P-value[a]
Deep breathing, heart rate	16.09 (7.31)	18.25 (8.44)	.085
Valsalva ratio, beats per minute	1.78 (0.67)	1.87 (0.42)	.233
Valsalva maneuver, end of phase 2 decline, mmHg	2.39 (14.22)	3.82 (9.97)	.424
Electrochemical skin conductance, μS	81.13 (13.44)	84.21 (9.89)	.084
Electrochemical skin conductance, μS/kg	1.22 (0.34)	1.21 (0.29)	.931
Epidermal nerve fiber density at proximal thigh, fibers/mm	13.96 (4.91)	13.40 (3.51)	.374
Epidermal nerve fiber density at calf, fibers/mm	10.10 (3.95)	9.67 (2.82)	.399
Sweat gland nerve fiber density at proximal thigh, fibers/mm	61.25 (12.56)	62.06 (13.17)	.776
Sweat gland nerve fiber density at calf, fibers/mm	55.94 (12.51)	55.49 (13.83)	.875
Heart rate supine, beats/minute	76.69 (13.06)	70.58 (14.48)	.006
Heart rate orthostatic, beats/minute	110.27 (18.14)	99.59 (15.96)	<.001
Systolic blood pressure supine, mmHg	118.27 (12.94)	116.27 (11.74)	.352
Systolic blood pressure orthostatic, mmHg	116.16 (14.75)	109.56 (12.62)	.007
Mean blood pressure supine, mmHg	89.81 (9.20)	87.68 (8.89)	.168
Mean blood pressure orthostatic, mmHg	91.54 (11.76)	86.51 (10.42)	.01
Diastolic blood pressure supine, mmHg	75.57 (8.11)	73.39 (8.33)	.112
Diastolic blood pressure orthostatic, mmHg	79.22 (11.29)	74.98 (9.70)	.023
Systolic CBFv supine, cm/sec	97.00 (18.29)	94.56 (14.13)	.415
Systolic CBFv orthostatic, cm/sec	78.63 (17.08)	81.10 (15.95)	.386
Mean CBFv supine, cm/sec	62.64 (12.41)	59.79 (8.89)	.076
Mean CBFv orthostatic, cm/sec	50.91 (11.75)	52.18 (11.53)	.517
Diastolic CBFv supine, cm/sec	45.47 (10.25)	42.43 (7.02)	.019
Diastolic CBFv orthostatic, cm/sec	37.03 (9.83)	37.73 (10.24)	.671
Mean CBFv orthostatic corrected for CO_2, cm/sec	60.52 (13.57)	61.29 (12.90)	.734
Maximal decline in orthostatic mean CBFv, cm/sec	17.08 (8.72)	13.68 (5.04)	<.001
Maximal decline in orthostatic mean CBFv, %	-26.75 (11.13)	-22.97 (8.51)	.014
Respiratory frequency supine, breaths per minute	15.91 (5.75)	14.80 (4.79)	.245
Respiratory frequency orthostatic, breaths per minute	16.04 (6.71)	13.29 (4.94)	.003
End-tidal CO_2 supine, mmHg	34.29 (3.80)	35.73 (3.91)	.025
End-tidal CO_2 orthostatic, mmHg	27.59 (6.39)	29.46 (4.68)	.027
Minimal end-tidal CO_2 orthostatic, mmHg	24.34 (6.47)	25.85 (5.32)	.157
Maximal decline in orthostatic end-tidal CO_2, mmHg	-9.95 (5.59)	-9.76 (5.32)	.838
Maximal decline in orthostatic end-tidal CO_2, %	-29.22 (16.29)	-27.13 (14.18)	.439
QASAT-CBFv, tilt response	6.34 (3.29)	4.71 (3.00)	.003
QASAT-ET-CO_2, tilt response	4.49 (3.81)	4.98 (4.13)	.456
QASAT-Autonomic failure	2.64 (2.09)	2.46 (2.11)	.607
QASAT-Cardiovagal	0.32 (0.62)	0.24 (0.62)	.451
QASAT-Adrenergic	0.86 (0.88)	0.85 (0.82)	.966
QASAT-Orthostatic tachycardia	5.08 (2.99)	4.44 (2.70)	.201
QASAT-Sudomotor	1.34 (1.69)	1.24 (1.50)	.729
QASAT-ENFD	0.76 (1.33)	0.51 (0.95)	.147

© 2025 Matthew GT et al. *JAMA Network Open*.

QASAT-SGNFD	0.19 (0.79)	0.25 (0.67)	.664

Abbreviations: CSS = Patients with central sensitization syndrome; non - CSS=patients without central sensitization syndrome; CBFv=cerebral blood flow velocity; QASAT = Quantitative Scale for Grading of Cardiovascular Autonomic Reflex Tests and Small Fibers from Skin Biopsies. Orthostatic results are reported at the 10th minute of the head-up tilt test. Results are presented as mean (SD). ENFD=epidermal nerve fiber density. SGNFD=sweat gland nerve fiber density. Data are mean±sd. [a] =Calculated using t test

© 2025 Matthew GT et al. *JAMA Network Open*.

eTable 2. Frequency of Abnormal Tests			
QASAT Variable	CSS (n=264)	Non - CSS (n=41)	P-value[a]
CBFv, reduced during the tilt, n, %	248 (93.9)	39 (95.1)	.999
ET-CO2, reduced during the tilt, n, %	191 (72.3)	28 (68.3)	.726
Autonomic failure, n, %	223 (84.5)	33 (80.5)	.676
Adrenergic, n, %	157 (59.5)	25 (61.0)	.991
Cardiovagal, n, %	69 (26.1)	7 (17.1)	.292
Orthostatic tachycardia, n, %	264 (100.0)	41 (100.0)	NA
Sudomotor, n, %	140 (53.0)	23 (56.1)	.843
ENFD, n, %	7 (63.6)	1 (50.0)	.999
SGNFD, n, %	18 (8.6)	5 (15.6)	.345
ENFD or SGNFD, n, %	102 (42.9)	15 (42.9)	.999
ENFD or SGNFD or Sudomotor, n, %	174 (68.2)	29 (74.4)	.559

Abbreviations: QASAT = Quantitative Scale for Grading of Cardiovascular Autonomic Reflex Tests and Small Fibers from Skin Biopsies. CSS=Patients with central sensitization syndrome; non - CSS=patients without central sensitization syndrome; SFN=small fiber neuropathy; ENFD=epidermal nerve fiber density. SGNFD=sweat gland nerve fiber density. n = number of patients with abnormal findings, %=percent of abnormal findings, [a]=Calculated using chi-squared test
