# Supplementary material for: Prevalence of Central Sensitization in Postural Tachycardia Syndrome
Source: JAMA Netw Open. 2026 Jan 13;9(1):e2553694. doi: 10.1001/jamanetworkopen.2025.53694 (PMC12801080; doi:10.1001/jamanetworkopen.2025.53694)
Supplement: Supplement 2. — Data Sharing Statement [file jamanetwopen-e2553694-s002.pdf]

Data Sharing Statement

Data
Data available: Yes
Data types: Deidentified participant data
How to access data: The data will be available from the corresponding author
When available: With publication

Supporting Documents
Document types: None

Additional Information
Who can access the data: researchers whose proposed use of the data has been approved
Types of analyses: for assessment of POTS in general
Mechanisms of data availability: with a signed data access agreement
